# Effect of treatment on urinary kidney injury molecule-1 in IgA nephropathy

**DOI:** 10.1186/1471-2369-14-139

**Published:** 2013-07-09

**Authors:** Mi Seon Seo, Moo Yong Park, Soo Jeong Choi, Jin Seok Jeon, Hyunjin Noh, Jin Kuk Kim, Dong Cheol Han, Seung Duk Hwang, So Young Jin, Soon Hyo Kwon

**Affiliations:** 1 Division of Nephrology, Seoul, South Korea; 2 Hyonam Kidney Laboratory, Soon Chun Hyang University Hospital, Seoul, South Korea; 3 Division of Nephrology, Soon Chun Hyang University Hospital, Bucheon, Korea; 4 Department of Pathology, Soon Chun Hyang University Hospital, Seoul, South Korea

**Keywords:** Biomarker, IgA nephropathy, KIM-1, Treatment in IgA nephropathy reduced the urinary KIM-1 excretion

## Abstract

**Background:**

Kidney injury molecule-1 (KIM-1) is a biomarker useful for detecting early tubular damage and has been recently reported as a useful marker for evaluating kidney injury in IgA nephropathy (IgAN). We therefore investigated whether treatment decreases urinary KIM-1 excretion in IgAN.

**Methods:**

We prospectively enrolled 37 patients with biopsy-proven IgAN. Urinary KIM-1 was assessed before and after treatment, which included low salt diet, blood pressure control, pharmacotherapy with angiotensin receptor blockers and/or angiotensin converting enzyme inhibitors, and immunosuppressive agents as necessary. The median treatment duration was 24 months.

**Results:**

Urinary KIM-1/creatinine (Cr) was significantly decreased in patients with IgAN after treatment compared to baseline (P < 0.0001, 1.16 [0.51-1.83] vs 0.26 [0.12-0.65] ng/mg). There was a decrease in the amount of proteinuria after treatment, but it was not statistically significant (P = 0.052, 748.1 [405-1569.7] vs 569.2 [252.2-1114] g/d). Estimated glomerular filtration rate (eGFR) did not change with treatment (P = 0.599, 79.28 ± 30.56 vs 80.98 ± 32.37 ml/min/1.73 m^2^). Urinary KIM-1 was not correlated with proteinuria baseline or follow up (pre-: R = - 0.100, P = 0.577, post-: R = 0.001, P = 0.993). In patients with higher baseline urinary KIM-1, both urinary KIM-1 level and proteinuria were significantly decreased following treatment.

**Conclusions:**

Treatment decreases urinary KIM-1/Cr in patients with IgAN. It also reduces proteinuria in patients with higher baseline urinary KIM-1. These results suggest a potential role for urinary KIM-1 as a biomarker for predicting treatment response in IgAN, however, further study is needed to verify this.

## Background

IgA nephropathy (IgAN) is the most common glomerulonephritis in the world, accounting for 20-45% of primary glomerular disease [1,2]. Long-term studies report that up to 30% of patients with IgAN progress to end-stage renal disease (ESRD) within twenty years [3-5]. Hypertension, massive proteinuria, elevated serum creatinine concentration, glomerular sclerosis, and interstitial fibrosis are predictors of poor renal outcome in IgAN [4,5]. However, these prognostic indicators have low sensitivity and specificity [6]. More accurate prognostic markers are required to predict the progress of IgAN and determine treatment.

Kidney injury molecule-1 (KIM-1) is a sensitive marker for detecting the presence of tubular damage [7-10]. KIM-1 expression is significantly induced in various primary and secondary kidney diseases and in allograft nephropathy [9-11]. Tubular KIM-1 expression is significantly associated with tubulointerstitial injury and inflammation, and increased urinary KIM-1 levels are strongly related to tubular KIM-1 expression [8,10,11]. Therefore, urinary KIM-1 is a valuable biomarker for the existence of tubulointerstitial damage.

Recent studies have shown that in patients with IgAN, urinary KIM-1 is closely associated with disease severity and is an independent predictor of ESRD [12,13]. However, it is still unclear whether urinary KIM-1 levels are affected by treatment. In the current study, we investigated whether urinary excretion of KIM-1 changes after treatment in patients with IgAN. We then further analyzed the relationship between urinary KIM-1 level and proteinuria.

## Methods

### Patients and methods

For the present study, prospective patients with biopsy-proven IgAN were enrolled from January 2009 at Soon Chun Hyang University Seoul and Bucheon Hospital. Study protocols were reviewed and approved by the Soon Chun Hyang University Seoul Hospital Institutional Review Board and Soon Chun Hyang University Bucheon Hospital Institutional Review Board, and written informed consent was obtained from each patient before enrollment. A diagnosis of IgAN was defined as the predominant mesangial deposition of IgA. Clinical and laboratory data were collected at the time of biopsy. Urinary samples were centrifuged at 3000 rpm for 10 min to remove cellular components, and the supernatant was frozen at -70°C until use. Urinary KIM-1 excretion was measured at diagnosis. Urinary KIM-1 was then measured at follow-up after about 2 years of treatment that included a low salt diet, blood pressure control, pharmacotherapy with angiotensin receptor blockers and/or angiotensin converting enzyme inhibitors, and immunosuppressive agents as necessary. All patients were treated with angiotensin receptor blockers (ARB) and/or angiotensin converting enzyme inhibitors (ACEi). Steroid pulse therapy and oral prednisolone was administered to patients with sustained massive protein excretion exceeding 2 g/day. Patients with other causes of IgA-positive glomerular staining (systemic lupus erythematosus, Henoch-Schönlein purpura, or liver disease) were excluded from the analysis.

### Detection of urinary KIM-1 by enzyme-linked immunossorbent assay

ELISA was performed in duplicate using a commercial kit (R&D System, MN, USA) in accordance with the manufacturer’s guidelines to measure KIM-1 protein levels in the urine. Inter- and intra-assay variability was <10%. To adjust urinary KIM-1 levels, urinary creatinine was also measured for each urine specimen. Adjusted urinary KIM-1 was expressed as urinary KIM-1concentration/creatinine concentration (ng/mg Cr). All KIM-1 levels in this study are expressed in this way. Reference values for urinary KIM-1/creatinine were obtained from urine measurements in 27 healthy volunteers who were all unmatched Korean men and women. Mean urinary KIM-1 excretion was 0.18 ng/mg with a 95% confidence interval (CI) of 0.096 to 0.27 ng/mg. The cutoff value for normal (0.27 ng/mg) was based on the upper limit of the 95% CI. The urinary KIM-1 of IgAN patients increased compared to that of normal volunteers [14].

### Grading of tubular atrophy/interstitial fibrosis

Grading of tubular atrophy/interstitial fibrosis followed the Oxford classification system, in which grading is determined by the percentage of cortical area involved by tubular atrophy or interstitial fibrosis, whichever is greater (tubular atrophy/interstitial fibrosis 0: 0-25%, tubular atrophy/interstitial fibrosis 1: 26-50%, tubular atrophy/interstitial fibrosis 2: > 50%) [15].

### Statistical analyses

Data are expressed as mean ± standard deviation or as median with interquartile range. Differences in quantitative data were evaluated using a paired *t*-test. Differences in qualitative data were evaluated using a chi-square test. When a normal distribution was present, ANOVA with a post hoc test or paired *t*-test were used. When an abnormal distribution was present, the nonparametric Kruskal-Wallis and Wilcoxon signed rank tests were used. Correlations between urinary KIM-1 and clinical parameters were determined using the Pearson correlation coefficient. P-values of <0.05 were considered statistically significant. Statistical analysis was performed using SPSS for Windows software version 14.0 (SPSS Inc., Chicago, IL, USA).

## Results

### Clinical and histopathological characteristics of patients with IgAN

Thirty-seven patients with IgAN were studied. Patient characteristics at baseline are presented in Table 1.

**Table 1:** **Patient characteristics at baseline**^**a**^

**Characteristic**	
Age (years)	39.18 ± 11.57
Male/Female (n){%}	18/19 {48.6/51.4}
Serum Creatinine (mg/dL)	1.24 ± 0.68
CKD EPI eGFR (ml/min/1.73 m^2^)	79.28 ± 30.56
24 hr Urinary Protein (mg/day)	748.1 [405-1569.7]
Mean Blood Pressure, systolic (mmHg)	125.4 ± 13.8
Mean Blood Pressure, diastolic (mmHg)	78.1 ± 8.7
Mesagial hypercellularity (%)	64.8
Segmental glomerulosclerosis (%)	91.8
Endocapillary hypercellularity (%)	37.8
Tubular atrophy/interstitial fibrosis 1 (%)	32.4
Tubular atrophy/interstitial fibrosis 2 (%)	21.6

^a^Data are expressed as mean ± SD or median with interquartile range.

### Treatment

During follow up, all patients were treated with ACEis and/or ARBs. Immunosuppressive therapy was initiated in five patients (13.5%) who either had progressive deterioration of renal function or persistent proteinuria (Table 2). A comparison of the clinical and histopathological characteristics of the patients receiving and not receiving immunosuppressive therapy is shown in Table 2. 24 hr urinary protein was significantly higher in the immunosuppressive therapy group (P < 0.01, 4.07 ±3.09 vs 1.00 ± 0.89 g/d). The proportion of patients with mesangial hypercellularity, segmental glomerulosclerosis, endocapillary hypercellularity, and tubular atrophy/interstitial fibrosis 2 was significantly higher in the immunosuppressive therapy group.

**Table 2:** **Clinical characteristics according to treatment modality**^**a**^

**Variable**	**Immunotherapy**	**No imunotherapy**	**P**
Age (years)	33.6 ± 18.32	40.06 ± 10.31	0.481
Male/Female {%}	4/1 {80/20}	15/17 {47/53}	<0.0001
Serum Creatinine (mg/dL)	1.52 ± 0.55	1.20 ± 0.69	0.347
CKD EPI eGFR (ml/min/1.73 m^2^)	70.06 ± 37.03	80.62 ± 29.90	0.505
24 hrs Urinary Protein (mg/day)	4077.18 ± 3098.19	1000.63 ± 899.85	<0.0001
Mean Blood Pressure, systolic (mm/Hg)	128 ± 13	125 ± 14.14	0.693
Mean Blood Pressure, diastolic (mmHg)	76 ± 8.3	78 ± 8.95	0.664
Mesagial hypercellularity (%)	100	59.4	<0.0001
Segmental glomerularsclerosis (%)	100	90.7	<0.0001
Endocapillary hypercellularity (%)	80	31.3	<0.0001
Tubular atrophy/interstitial fibrosis 1 (%)	0	37.5	<0.0001
Tubular atrophy/interstitial fibrosis 2 (%)	60	15.7	<0.0001
Baseline KIM-1/Cr (ng/mg)	0.51 [0.31-1.23]	1.12 [0.62-2.02]	0.155
Follow-up KIM-1/Cr (ng/mg)	0.52 [0.01-0.64]	0.26 [0.13-0.73]	0.625
Δ KIM-1/Cr (ng/mg)	0.49 [0.34-0.71]	0.81 [0.17-1.23]	0.230
Baseline eGFR (ml/min/1.73 m^2^)	70.65 ± 37.03	80.62 ± 29.9	0.505
Follow-up eGFR (ml/min/1.73 m^2^)	72.82 ± 48.44^*^	82.26 ± 30.03^**^	0.552
Baseline proteinuria (mg/d)	4855.1 [1569.7-5560.5]	720 [401-1189.7]	
Follow-up proteinuria (mg/d)	1323.9 [374.6-1480]	545.6 [250.5-824]	
P	0.273	0.077	

^a^Data are expressed as median ± SD or median with interquartile range.

Compared with baseline eGFR: ^*^P = 0.904; ^**^P = 0.572.

### Concentration of urinary KIM-1 at baseline and follow-up

**Figure 1:**
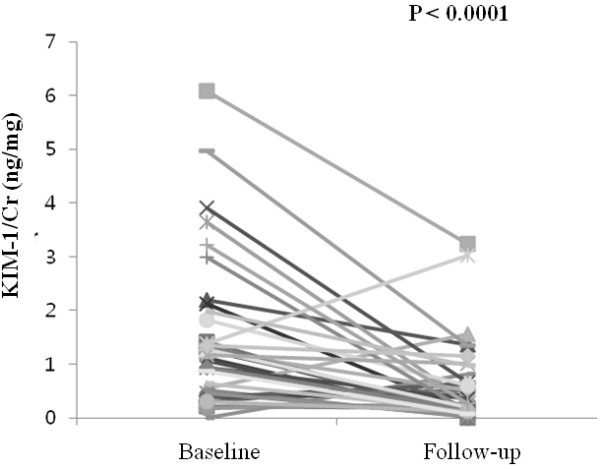
Comparison of baseline and follow-up urinary KIM-1/Cr (ng/mg).

The mean follow up duration was 23.56 ± 5.08 months. Baseline urinary KIM-1 was 1.16 [0.51-1.83] ng/mg while the follow-up level was 0.26 [0.12-0.65] ng/mg. Urinary KIM-1 was significantly decreased in patients with IgAN after treatment (Figure 1). KIM-1 levels normalized in 19 patients (51.35%) after treatment.

### Clinical parameters of IgAN patients at baseline and follow-up

At follow-up, there was a decrease in the amount of proteinuria, but it was not statistically significant (P = 0.052, 748.1 [405-1569.7] vs 569.2 [252.2-1114] g/d). Treatment did not affect estimated glomerular filtration rate (eGFR) (P = 0.599, 79.28 ± 30.56 vs 80.98 ± 32.37 ml/min/1.73 m^2^) (Table 3).

**Table 3:** **Comparison of baseline and follow-up clinical parameters**^**a**^

**Variable**	**Baseline**	**Follow-up**	**P**
Serum Creatinine (mg/dL)	1.0 [0.9-1.3]	1.0 [0.8-1.3]	0.542
CKD EPI eGFR (ml/min/1.73 m^2^)	79.28 ± 30.56	80.98 ± 32.37	0.599
SBP (mmHg)	125 ± 13.86	119 ± 15.97	0.050
DBP (mmHg)	78 ± 8.76	74 ± 10.68	0.080
24 hrs Urinary Protein (mg/day)	748.1 [405-1569.7]	569.2 [252.2-1114]	0.052

^a^Data are expressed as median ± SD or median with interquartile range.

Although we found that proteinuria was not significantly reduced after treatment, this was not the case when we analyzed treatment response in patients stratified according to tertiles of baseline urinary KIM-1. After treatment, urinary KIM-1 and proteinuria were significantly decreased in patients in the highest tertile (urinary KIM-1/Cr: P = 0.008, proteinuria: P = 0.01) (Table 4).

**Table 4:** **Treatment response stratified by baseline urinary KIM-1 level**^**a**^

	**n = 13**	**n = 12**	**n = 12**
Baseline KIM-1/Cr (ng/mg)	0.39 [0.3-0.51]	1.19 [1.02-1.28]	2.59 [1.94 -3.7]
Follow-up KIM-1/Cr (ng/mg)	0.26 [0.12-0.61]	0.17 [0.09-0.4]	0.62 [0.22-1.35]
P	0.91	0.002	0.008
Baseline proteinuria (mg/d)	1109 [528.2-1639]	537.5 [296.9-1453.2]	916.5 [508.2-1262.2]
Follow-up proteinuria (mg/d)	722.1 [252.2-1323.9]	509.5 [237.2-979.6]	518.3 [361.8-766]
p	0.333	0.678	0.018

^a^Data are expressed as median with interquartile range.

When comparing patients whose KIM-1 levels did and did not normalize following treatment, there was no significant decrease of proteinuria or eGFR in either group. There was also no significant difference in follow-up proteinuria or eGFR between the two groups (Table 5). We also compared proteinuria and eGFR between patients who did and did not receive immunosuppressive therapy. There was no significant decrease in proteinuria or eGFR in either group after treatment (Table 2).

**Table 5:** **Baseline and follow-up clinical parameters according to normalization of urinary KIM-1**^**a**^

	**Patients with normalization (n = 19)**	**Patients without normalization (n = 18)**	**P**
Baseline proteinuria (mg/d)	718 [348.3–1417]	1006.25 [537.1-2269.9]	
Follow-up proteinuria (mg/d)	522 [868.4-1114]^*^	572.65 [270-1260.7]^**^	0.871
Baseline eGFR (ml/min/1.73 m^2^)	88.6 ± 36.2	72.6 ± 29.4	
Follow-up eGFR (ml/min/1.73 m^2^)	78.3 ± 34^#^	83.8 ± 31.2&	0.283

^a^ Data are expressed as median ± SD or median with interquartile range.

Compared with baseline proteinuria: ^*^P = 0.925; ^**^P = 0.11.

Compared with baseline eGFR: ^#^P = 0.334; &P = 0.141.

Finally, we analyzed proteinuria and eGFR according to reduction of 24 hr urinary protein. There was no significant difference in baseline or follow-up KIM-1 between patients whose 24 hr urinary protein did and did not decrease by more than 50%. However, there was a significant difference in follow-up eGFR between the two groups (P = 0.04) (Table 6).

**Table 6:** **Baseline and follow-up clinical parameters according to reduction in 24 hr urinary protein**^**a**^

**Variable**	**Proteinuria reduction > 50% (n = 10)**	**Proteinuria reduction < 50% (n = 26)**	**P**
Baseline KIM-1/Cr (ng/mg)	1.17 [0.44-1.91]	1.16 [0.57-1.62]	0.811
Follow-up KIM-1/Cr (ng/mg)	0.41 [0.21-0.74]	0.19 [0.11-0.64]	0.393
Baseline eGFR (ml/min/1.73 m^2^)	87.86 ± 30.34	76.1 ± 30.59	0.305
Follow-up eGFR (ml/min/1.73 m^2^)	98.78 ± 27.86	74.39 ± 31.87	0.040

^a^Data are expressed as median ± SD or median with interquartile range.

In our study, baseline urinary KIM-1 was not correlated with renal KIM-1 grade or Oxford classification of renal damage [14].

### Association between urinary KIM-1 and proteinuria

Urinary KIM-1 was not correlated with proteinuria at baseline or follow-up (baseline: R = 0.100, P = 0.577, follow-up: R = 0.001, P = 0.993).

## Discussion

Interstitial kidney damage is a consistent predictor of renal prognosis. Several studies have identified clinical features and histological findings which serve as predictors of adverse renal outcomes. In particular, tubulointerstitial changes have been recognized as important prognostic factors in IgAN [16-19]. However, tubulointerstitial damage is not always associated with proteinuria, which is a marker of glomerular damage [20]. Therefore, it is important to identify a biomarker specific for tubular damage to accurately monitor therapy response and tubular damage in IgAN.

Our data showed that IgAN treatment significantly decreased urinary KIM-1 concentration. This finding suggests that urinary KIM-1 may be a useful biomarker for evaluating and predicting treatment response. Previous studies have found that KIM-1 expression in proximal tubules is associated with interstitial fibrosis and inflammation in various renal diseases [10,11]. Several studies have reported that urinary KIM-1 levels are correlated with tubular KIM-1 expression in experimental models and human renal disease [8-10,21]. Because of this, and because it is a non-invasive marker for kidney damage, KIM-1 is a promising biomarker. In IgAN, urinary KIM-1 has clinical implications in predicting renal outcome and assessing tubulointerstitial damage [12,13]. Waanders et al. have reported that urinary KIM-1 levels decreased following antiproteinuric treatment in non-diabetic patients. However, this study included only a small number of IgAN patients (n = 5) and had a relatively short follow up duration (6 weeks) [20]. Because IgAN is the most common glomerulonephritis in the world, KIM-1 should be verified as a therapeutic parameter with a long-term study in a large IgAN cohort.

We treated biopsy-proven IgAN patients using conventional methods for 24 months, which resulted in a decrease in KIM-1. We believe there are several possible mechanisms for this result. First, treatment can reverse interstitial damage. All patients were treated with at least one RAS antagonist. One observational human study reported that treatment with an ARB can improve renal interstitial fibrosis. In that study, patients with biopsy-proven IgAN and non-IgA mesangioproliferatve glomerulonephritis were treated with an ARB for an average of 28 months. Although the global sclerosis ratio was not significantly altered by treatment with an ARB, 13 of the 15 patients showed decreases in mesangial matrix and interstitial fibrosis [22]. Kramer at al. showed treatment with an ARB reversed tubular Kim-1 expression in an adriamycin induced nephropathy model [21]. In several other experimental models, ARBs produced renal structural improvement [23-25]. These studies suggest that RAS blockade can improve tubulointerstital damage and decrease urinary Kim-1, a marker of tubular damage. Another explanation for the decreased KIM-1 is that a reduction in proteinuria can lower urinary KIM-1 excretion. A recent study showed that tubular expression of Kim-1 decreased in proportion to decreases in proteinuria as a result of RAAS blockade in adriamycin induced nephropathy and homozygous Ren2rats [21,26]. Another study found proteinuria was able to cause tubulointerstital injury [27]. These studies support the hypothesis that decreased proteinuria reduces proteinuria-induced tubular damage and decreases Kim-1 expression.

A previous study involving patients with various renal diseases showed that even when proteinuria was reduced below a certain threshold (less than 1 g/d), urinary KIM-1 did not normalize in 14 of 16 patients [20]. Although our treatment did not profoundly decrease proteinuria, KIM-1 did reach the normal range in 50% of patients. In patients in the highest tertile for baseline KIM-1, urinary KIM-1 and proteinuria were significantly decreased after treatment. Previous studies reported Kim-1 was not detected in severely damaged tubules [10,14,21]. As tubular KIM-1 expression is specific to ongoing tubular cell damage and differentiation [28,29], we hypothesized urinary KIM-1 represented reversibility. In an experimental model, Kim-1 expression was reduced in proportion to proteinuric reduction [21]. This supports the potential of KIM-1 as a biomarker for tubulointerstitial damage and repair.

In our study, treatment reduced urinary KIM-1, but this was not accompanied by a reduction in other clinical parameters. The progression of IgAN is slow, and the response to treatment is also slow. In this study, the follow-up period was relatively short. The proteinuria reduction for all patients did not reach statistical significance, however, it showed a trend in that direction (p = 0.053). The antecedent reduction of urinary KIM-1 suggests that urinary KIM-1 may be a more sensitive biomarker and has unique clinical implications in IgAN. KIM-1 has been reported as a better independent prognostic marker than proteinuria for IgAN [12]. Although proteinuria has been considered a main risk factor for decreased kidney function, this is an inconsistent conclusion [6,30,31]. Therefore, long-term studies are needed to evaluate whether treatments targeting KIM-1 can improve outcomes in patients with IgAN.

The correlation between urinary KIM-1 and proteinuria is also inconsistent [8,10,32]. Differences in urinary KIM-1 measurement methods may be cause this discrepancy. Preclinical KIM-1 assays should be validated in a large cohort and a variety of clinical settings. Recently, a number of urinary biomarkers have emerged for kidney disease. These urinary biomarkers have the potential to facilitate early diagnosis and management of kidney damage. They may be able to alert clinicians to kidney damage before it can be identified using conventional measurements, such as change in glomerular filtration rate or proteinuria. It is therefore necessary to verify the usefulness of KIM-1 by conducting a long-term study in a large population. This will determine whether treatments targeting KIM-1 can improve outcome in patients with chronic kidney disease.

This study had several limitations. First, it describes a small number of patients who have well preserved renal function. This population does not represent the various stages of IgAN. Second, the ELISA method used in our study has limited sensitivity. However, the sensitivity was sufficient to investigate the association between urinary KIM-1 and clinical parameters in patients with IgAN. Lastly, because of difficulties in obtaining repeat biopsies, we cannot conclude that decreases in the concentration of urinary KIM-1 improve renal outcomes in IgAN.

## Conclusions

Treatment decreases urinary KIM-1/Cr in patients with IgAN. Urinary KIM-1 does not correlate with proteinuria. Treatment reduced proteinuria in patients in the highest tertile for baseline KIM-1. Future studies should be undertaken to verify the usefulness of KIM-1 as biomarker in IgAN.

## Competing interest

The authors have no competing interests.

## Authors’ contribution

KSH was the principal investigator; participated in design and coordination, and helped to draft the manuscript. SMS participated in coordination, wrote the draft manuscript. PMY participated in design and manage the research porject, CSJ participated in design, helped to collect sample and analyzed statistic data. JJS carried measurement of urinary KIM-1. NHJ participated in study design, enrolled patients, and managed samples. HSD, KJK, HDC enrolled patients at clinical office and interpretation of data and drafting and revising the manuscript. JSY made the pathological confirmations of IgAN, reviewed medical records, and participated in data analysis. All authors read and approved the final manuscript.

## Pre-publication history

The pre-publication history for this paper can be accessed here:

http://www.biomedcentral.com/1471-2369/14/139/prepub
